# Lenticular mitoprotection. Part A: Monitoring mitochondrial depolarization with JC-1 and artifactual fluorescence by the glycogen synthase kinase-3β inhibitor, SB216763

**Published:** 2013-06-27

**Authors:** Morgan M. Brooks, Sudha Neelam, Rafal Fudala, Ignacy Gryczynski, Patrick R. Cammarata

**Affiliations:** 1 Department of Cell Biology and Anatomy, University of North Texas Health Science Center at Fort Worth, Fort Worth, TX; 2 Department of Molecular Biology and Immunology, University of North Texas Health Science Center at Fort Worth, Fort Worth, TX

## Abstract

**Purpose:**

Dissipation of the electrochemical gradient across the inner mitochondrial membrane results in mitochondrial membrane permeability transition (mMPT), a potential early marker for the onset of apoptosis. In this study, we demonstrate a role for glycogen synthase kinase-3β (GSK-3β) in regulating mMPT. Using direct inhibition of GSK-3β with the GSK-3β inhibitor SB216763, mitochondria may be prevented from depolarizing (hereafter referred to as mitoprotection). Cells treated with SB216763 showed an artifact of fluorescence similar to the green emission spectrum of the JC-1 dye. We demonstrate the novel use of spectral deconvolution to negate the interfering contributing fluorescence by SB216763, thus allowing an unfettered analysis of the JC-1 dye to determine the mitochondrial membrane potential.

**Methods:**

Secondary cultures of virally transfected human lens epithelial cells (HLE-B3) were exposed to acute hypoxic conditions (approximately 1% O_2_) followed by exposure to atmospheric oxygen (approximately 21% O_2_). The fluorescent dye JC-1 was used to monitor the extent of mitochondrial depolarization upon exposure of inhibitor treatment relative to the control cells (mock inhibition) in atmospheric oxygen. Annexin V-fluorescein isothiocyanate/propidium iodide staining was implemented to determine cell viability.

**Results:**

Treatment of HLE-B3 cells with SB216763 (12 µM), when challenged by oxidative stress, suppressed mitochondrial depolarization relative to control cells as demonstrated with JC-1 fluorescent dye analysis. Neither the control nor the SB216763-treated HLE-B3 cells tested positive with annexin V-fluorescein isothiocyanate/propidium iodide staining under the conditions of the experiment.

**Conclusions:**

Inhibition of GSK-3β activity by SB216763 blocked mMPT relative to the slow but consistent depolarization observed with the control cells. We conclude that inhibition of GSK-3β activity by the GSK-3β inhibitor SB216763 provides positive protection against mitochondrial depolarization.

## Introduction

The loss of cellular respiration increases the levels of reactive oxygen species (ROS) in human lens epithelial cells (HLE-B3) [1]. A sufficient increase in ROS may also cause a collapse of the mitochondrial membrane potential (∆Ψ) in a process termed mitochondrial membrane permeability transition (mMPT) [2,3]. Dissipation of ∆Ψ prompts further disruption of the electron transport chain, decreasing the production of ATP, and increasing the formation of ROS [4,5] in a harmful downward cycle. The loss of ∆Ψ also leads to the release of apoptotic factors that can cause cellular dysfunction and cell death [6].

HLE-B3 cells have developed protective mechanisms to prevent the loss of ∆Ψ caused by mMPT during oxidative stress. mMPT is mediated via the opening of the mitochondrial permeability transition pore, a pore permeable to solutes of less than 1.5 kDa and sensitive to an accumulation of ROS [7-9]. Studies in the cardioprotection literature have shown that GSK-3β is a crucial enzyme involved in preventing the collapse of ∆Ψ through dynamic regulation of the opening and closing of the mitochondrial permeability transition pore [10,11]. Active GSK-3β allows the pore to open whereas the enzyme’s inactivation blocks the pore from opening. Mitochondrial protection (hereafter referred to as mitoprotection) dictates that impeding the opening of the permeability transition pore prevents mitochondrial depolarization, thus averting entry into the cell death pathway [12]. Recent studies involving pre-/post-conditioning ischemic reperfusion in mouse and rat cardiac myocytes have shown that under conditions of oxidative stress, inhibition of GSK-3β activity prevents the loss of ∆Ψ [12-14]. To date, no such studies linking GSK-3β with mMPT have been reported in an ocular system.

Currently, there have been no reported studies involving the use of SB216763 (a GSK-3β inhibitor) as it influences the mitochondrial membrane potential as analyzed with the potentiometric dye JC-1. In this report, we show that SB216763 contributes to the green emission spectrum thus contributing to a false result of depolarization. Our study describes the use of a technique that will enable the precise reconvolution of the proper contributions from JC-1 green and red emissions. The data demonstrate that preventing mitochondrial depolarization, via the use of SB216763, presumably due to blocking the opening of the mitochondrial membrane permeability transition pore, positively correlates with inhibiting GSK-3β enzymatic activity.

The influence of SB216763 on the pore as analyzed with JC-1 analysis has not previously been reported due to the emission spectrum of cells treated with SB216763 in the absence of JC-1 revealing a broad-spectrum over the range of 500–650 nm. In this study, we make novel use of spectral deconvolution based on experimental measurements, fluorophore reference spectra, and an algorithm for least-squares minimization to produce corresponding unmixed spectra. After deconvolution, the green/red intensity ratios (540/595 nm) provided by the JC-1 dye may be used to calculate the extent of mitochondrial depolarization.

## Methods

### Materials

Glycogen synthase kinase inhibitor 3-(2,4-dichlorophenyl)-4-(1-methyl-1H-indol-3-yl)-1H-pyrrole-2,5-dione (SB216763) was purchased from Sigma-Aldrich (St. Louis, MO). The stock inhibitor was prepared by adding dimethyl sulfoxide (DMSO) to make 16 mM of SB216763. The mitochondrial dye 1H-benzimidazolium-5,6-dichloro-2-[3-(5,6-dichloro-1,3-diethyl-1,3-dihydro-2H-benzimidazol-2-ylidene)-1-propenyl]-1,3-diethyl-iodide (JC-1) was obtained from Life Technologies (Grand Island, NY). All other reagents were acquired from other commercially available sources as previously reported [2].

### Cell cultures

HLE-B3 cells, a human lens epithelial cell line immortalized by the SV-40 virus [15], were obtained from U. Andley (Washington University School of Medicine, Department of Ophthalmology, St. Louis, MO). The cells were maintained in minimal essential media (MEM) containing 5.5 mM glucose supplemented with 20% fetal bovine serum (Gemini Bio-Products, Sacramento, CA), 2 mM L-glutamine, nonessential amino acids, and 0.02 g/L gentamycin solution (Sigma-Aldrich) and cultured at 37 °C and 5% CO_2_ to 95% O_2_ [2]. All experiments were performed using monolayers of HLE-B3 cells that did not exceed passage 25. Cells were sub-cultured 4 to 5 days before the experiment and placed in MEM containing 20% fetal bovine serum. On the day of the planned experiment, cells were washed in serum-free MEM and then switched to serum-free MEM.

### JC-1 fluorescence analysis and confocal microscopy

After the HLE-B3 cells were subjected to inhibitor treatments, the cells were stained with JC-1 to determine the mitochondrial membrane potential. JC-1 is a membrane-permeable lipophilic dye that exists as J-aggregates in the mitochondrial matrix (red fluorescence) and as monomers in the cytoplasm (green fluorescence). During mitochondrial depolarization, the red J-aggregates form green monomers due to a change in ∆Ψ [16]. Thus, depolarization can be measured as an increasing green fluorescent/red fluorescent intensity ratio.

The JC-1 assay was performed as follows. HLE-B3 cell monolayers were maintained in serum-free MEM with or without inhibitor treatment, brought through ambient oxygen into hypoxia, and then later switched back to ambient oxygen as described above. At the end of the hypoxic exposure, the hypoxic media on cells (oxygen depleted) were poured off, and fresh (oxygen rich) serum-free MEM (with or without an inhibitor) containing 5 µg/ml JC-1 was added for 30 min in a tissue culture incubator. The stained HLE-B3 cells were then rinsed twice using serum-free MEM, and fresh oxygenated serum-free MEM (with or without inhibitor, but no JC-1 dye) was added. After the fresh media was added, the cells were analyzed with a Cary Eclipse spectrofluorometer (Varian Inc., Belrose, Australia).

### Emission spectrum analysis

Fluorescence emission spectra were collected in 15 min intervals using a Cary Eclipse spectrofluorometer. Measurements were performed in front-face mode using secondary cultures of HLE-B3 cells on coverslips in the presence of an inhibitor or DMSO. The JC-1 emission spectrum was measured with the excitation at 470 nm. To analyze the ratiometric changes of the JC-1 spectrum, we performed spectral deconvolution with Mathcad software (Parametric Technology Corp., Needham, MA). The deconvolution was based on experimental measurements, fluorophore reference spectra, and an algorithm for least-squares minimization to produce corresponding unmixed spectra in graph form with error provided in minimal least-squares values for flexibility in analysis. After deconvolution, the green fluorescent/red fluorescent intensity ratios (540/595 nm) were calculated to determine mitochondrial depolarization.

### Annexin V-fluorescein isothiocyanate/propidium iodide apoptosis detection assay

To evaluate cell viability, a Clontech Apoalert annexin V apoptosis assay (Mountain View, CA) was used in conjunction with confocal microscope imaging to monitor apoptosis and necrosis. HLE-B3 cells were incubated for 90 min with serum-free MEM containing SB216763 (12 µM) or 0.05% DMSO vehicle. After the incubation period, the cells were switched to hypoxia for 3 h. At the end of the hypoxic exposure, the oxygen-depleted media was removed, and fresh, oxygenated serum-free MEM containing either SB216763 or DMSO control were added for 60 min in atmospheric oxygen. At the end of this 60 min period, half the cell samples were stained with annexin V-fluorescein isothicyanate/propidium iodide by removing the culture media, and washed one time with the blocking buffer provided in the Apoalert annexin V apoptosis detection kit (Clontech). After the wash, the samples had blocking buffer containing 50 ng/ml annexin V-fluorescein isothiocyanate and 125 ng/ml propidium iodide added to the dishes for 15 min in the dark at room temperature. The blocking buffer was subsequently removed, and fresh serum-free MEM containing SB216763 or DMSO were added. The samples not stained with annexin V-fluorescein isothicyanate/propidium iodide remained in serum-free MEM containing either SB216763 or DMSO throughout the washing and 15 min staining period. Random samples were imaged on a Zeiss LSM 410 confocal microscope (Peabody, MA). Red fluorescence indicates staining of DNA with propidium iodide, and, in combination with the green annexin V fluorescence, suggests a loss of plasma membrane integrity typical of necrotic cell death [17].

## Results

### SB216763 inhibits the enzymatic activity of glycogen synthase kinase-3β and prevents mitochondrial depolarization during oxidative stress

**Figure 1:**
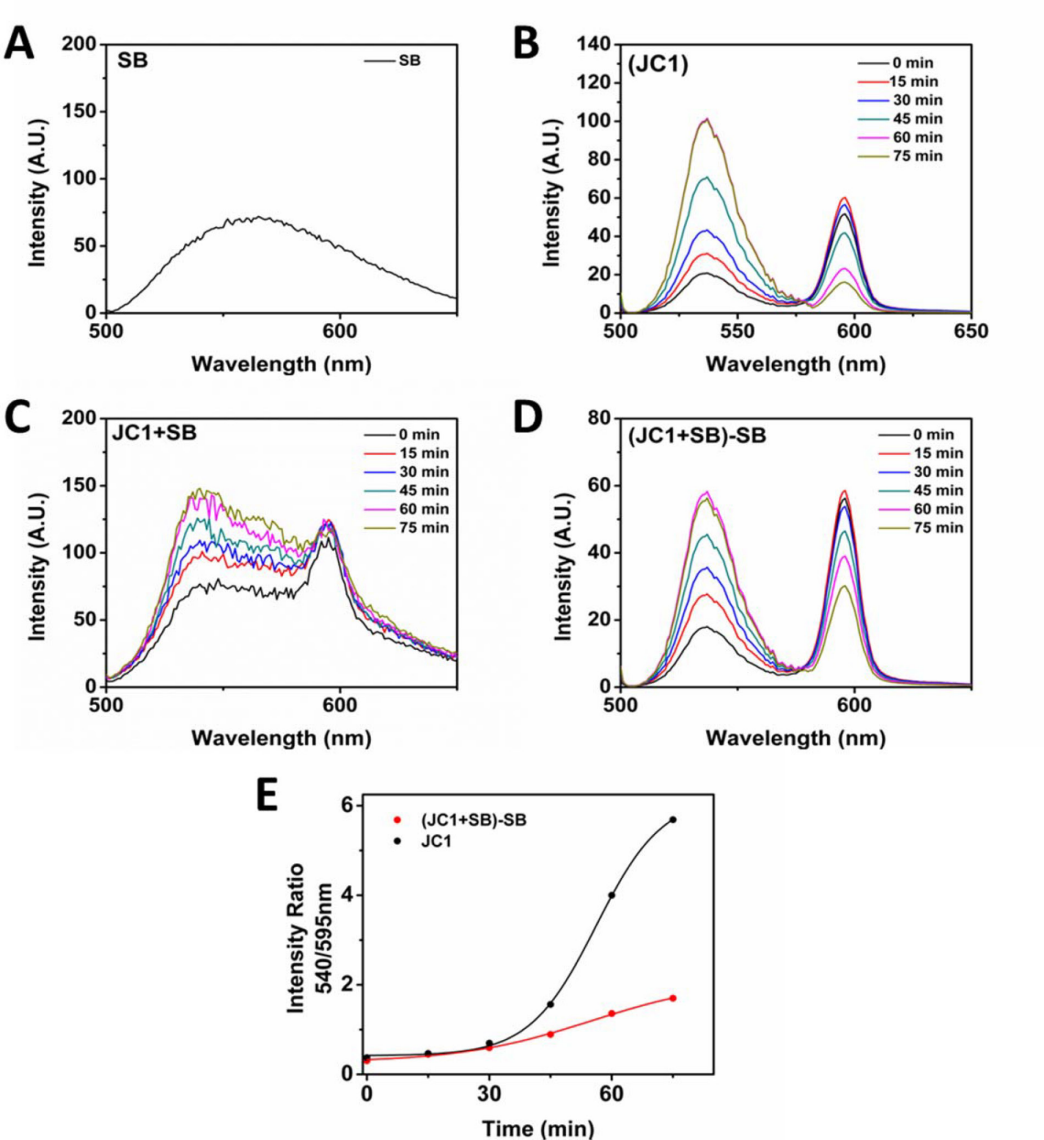
Emission spectrum analysis of mitochondrial depolarization in HLE-B3 cells treated with SB216763. HLE-B3 cells were incubated for 90 min with serum-free minimal essential medium (MEM) containing SB216763 (12 µM) or 0.05% DMSO vehicle. After the incubation period, the cells were switched to hypoxia for 3 h. At the end of the hypoxic exposure the oxygen depleted media was removed and fresh, oxygenated serum-free MEM containing 5µg/ml JC-1, a depolarization sensitive dye, with either SB216763 or DMSO was added for 30 min in atmospheric oxygen. At the end of the 30 min period, the culture media was again removed and fresh serum-free MEM containing SB216763 or DMSO added. A random field of cells was scanned every 150 sec for 75 min. **A**: The emission spectra for HLE-B3 cells treated with 12 µM SB216763 was analyzed. **B**: The analysis of the serial emission spectrum for HLE-B3 cells treated with 0.05% DMSO in the presence of the potentiometric dye (JC-1). **C**: The analysis of the serial emission spectrum of HLE-B3 cells treated with 12 µM SB216763 and JC-1. **D**: The analysis of the emission spectra from the SB216763 treated cells (refer to **C**) after deconvolution was used to suppress the contribution from the inhibitor (refer to **A**). Emission spectrum analysis occurred every 150 sec over 75 min but only the data points for every 15 min interval is shown. **E**: The graph of green emission (540 nm) over red emission (595 nm) of SB216763 treated cells with JC-1 after deconvolution (red dots) versus DMSO with JC-1control cells (black dots). The experiment was performed on three independent populations of lens epithelial cells. The data is not a composite of the three runs but rather the best of the three is shown.

The SB216763-treated and DMSO mock-treated cells were monitored for mMPT using the JC-1 dye. The basis of the JC-1 depolarization assay is to monitor the shift from red fluorescence to green fluorescence caused by the conversion of J-aggregates in the mitochondrial matrix (red) to J-monomers dispersing in the cytosol (green) upon opening of the membrane permeability transition pore. The emission spectrum of the cells treated with SB216763 in the absence of JC-1 revealed a broad-spectrum over the range of 500–650 nm (Figure 1A). The emission spectra of the JC-1-treated cells treated with DMSO consisted of two, well-separated spectra: green with a maximum at 540 nm and red with the maximum at 595 nm (Figure 1B). Cells stained with JC-1 and jointly treated with SB216763 displayed a broad-spectrum fluorescence because the green fluorescence contributed by the inhibitor overlapped with the green and red fluorescence of the JC-1 dye, distorting the green peak that skewed the green/red (G/R) ratio (Figure 1C). The distortion caused by the SB216763 fluorescence was subtracted from the total spectrum using spectral deconvolution. As stated in the Methods section, deconvolution was achieved by using the fluorophore reference spectra in Figure 1A and an algorithm for least-squares minimization. The deconvolution produced corresponding spectra with clear green (540 nm) and red (595 nm) maximums that were used to calculate the G/R ratio (Figure 1D). Analysis of the G/R ratio revealed that SB216763-treated cells had substantially suppressed mitochondrial depolarization relative to the controls (Figure 1E).

### SB216763 does not affect cell viability

**Figure 2:**
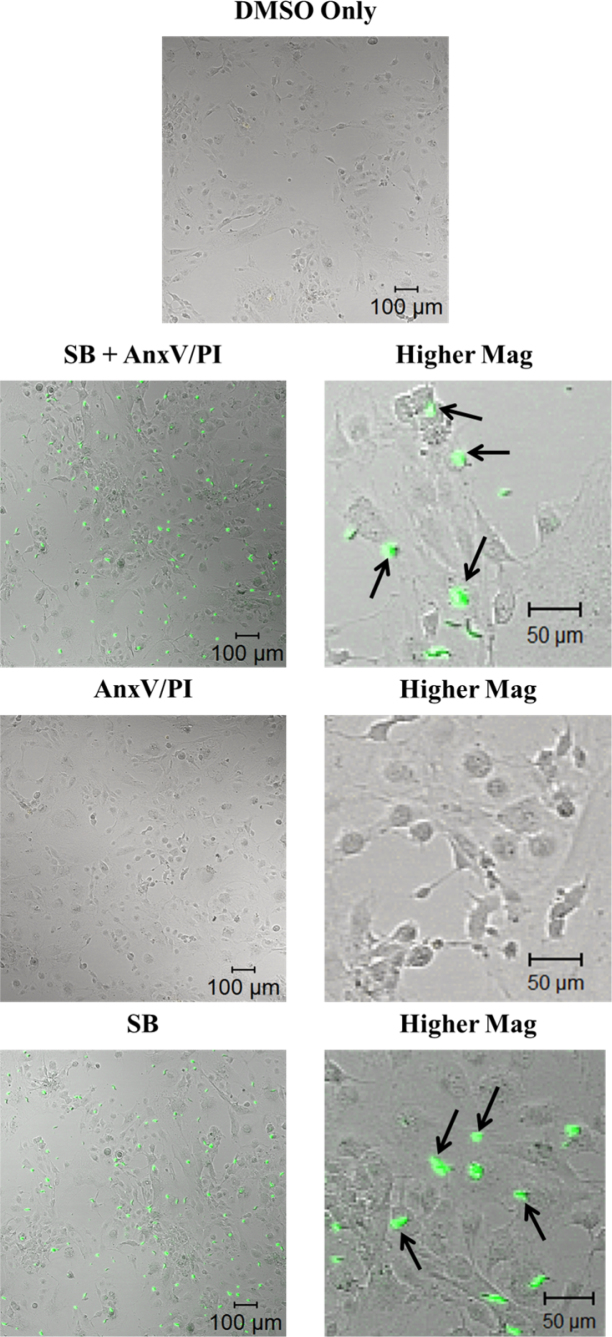
Annexin V-fluorescein isothicyanate/propidium iodide analysis of apoptosis in HLE-B3 cells treated with SB216763. Cells were treated with annexin V-fluorescein isothicyanate/propidium iodide either in the presence or absence of SB216763. Top row: DMSO control cells without annexin V-fluorescein isothicyanate/propidium iodide is shown. Second row: (left panel) Image of SB216763 treated cells additionally stained with annexin V-fluorescein isothicyanate/propidium iodide; (right panel) higher magnification. Third row: (left) Image of cells stained with annexin V-fluorescein isothicyanate/propidium iodide only; (right) higher magnification. Bottom row: (left panel) Image of SB216763 treated cells; (right panel) higher magnification. Black arrows indicate false fluorescence from undissolved particulate SB216763.

To determine the effect of inhibition of GSK-3β activity by treatment with SB216763 on cell viability, an annexin V-fluorescein isothicyanate/propidium iodide assay was implemented under similar conditions as the JC-1 analysis. Mock-treated cells (DMSO control) not treated with SB216763 or stained with annexin V-fluorescein isothicyanate/propidium iodide are shown in the top row of Figure 2. The SB216763-treated cells stained with annexin V-fluorescein isothicyanate/propidium iodide (Figure 2, second row) showed a lack of red cells with green halo, indicating no apoptosis/necrosis. (Fluorescent green staining of the plasma membrane indicates apoptosis by the release of annexin V to the outer leaflet of the plasma membrane. Red staining of DNA with propidium iodide, in conjunction with green annexin V staining, indicates a loss of plasma membrane integrity typical of necrotic cells) [17]. However, artifactual SB216763 fluorescence resulting from undissolved particulate matter was noted. Cells treated with annexin V-fluorescein isothicyanate/propidium iodide alone confirmed the lack of red/green fluorescence (Figure 2, third row). The SB216763-treated cells in the absence of annexin V-fluorescein isothicyanate/propidium iodide corroborated the fact that SB216763 displays artifactual fluorescence (Figure 2, compare the second row to the bottom row).

## Discussion

The dynamics of the mitochondrial membrane permeability transition pore and its regulation regarding the opening and closing of the pore are tightly controlled by GSK-3β [10-12,18]. SB216763 is a potent and highly selective inhibitor of GSK-3β activity [19]. A role for GSK-3β in mitoprotection has been supported by the work of Juhaszova et al. [12], who concluded that “this enzyme, located proximally (in the sense of a signaling cascade) to the mitochondrial transition pore complex, acts as a master switch to convey a multiplicity of protective signals to their final point, the mitochondrial permeability transition pore.” Forster et al. [14] demonstrated that inhibition of GSK-3β by SB216763 prevented the oxidant-induced depolarization of ∆Ψ (presumably caused by the inhibition of the opening of the transition pore) in rat ventricular myocytes. The reintroduction of atmospheric oxygen after acute hypoxia led to a slow but inevitable loss of ∆Ψ (Figure 1E). SB216763 was used to demonstrate that inactivation of the enzyme prevents the loss of membrane potential (Figure 1E).

Interpretations other than inhibiting GSK-3β resulting in preventing the opening of the mitochondrial permeability transition pore are equally plausible as an explanation for our data. JC-1 is a novel membrane-permeable cationic carbocyanine dye that accumulates to a high concentration in the mitochondria as J-aggregates and exhibits an emission maximum at about 590 nm. JC-1 may diffuse across the outer and inner mitochondrial membrane in response to the changes in the mitochondrial membrane potential. Diffusion to the cytoplasm from the mitochondria results in a lower concentration, where the dye exists as a monomer and yields green fluorescence with an emission maximum about 540 nm. Loss of JC-1 from the mitochondria is not inevitably a measure of opening of the membrane permeability transition pore. Rather, the loss is a measure of the decrease in the inner mitochondrial membrane potential, possibly due to the loss of integrity of the inner and outer mitochondrial membranes. That is, JC-1 is permeable to the mitochondrial membrane, independent of the transition pore, and the dye’s accumulation or loss does not necessarily depend on the state of the transition pore. Therefore, treatment with SB216763 might alternatively be explained as the drug’s ability to maintain the transmembrane potential of the inner mitochondrial membrane. Regardless of how one chooses to interpret the data, the fact remains that SB216763 elicited lenticular mitoprotection.

Past studies have suggested that the phosphorylation of GSK-3β indicates inactivation of the enzyme [20]. Therefore, a key question that we wished to address in our study was whether the presumed inhibition of GSK-3β activity would positively correlate with the prevention of opening the mitochondrial membrane transition pore. To evaluate whether SB216763 blocked mitochondrial depolarization, we performed a JC-1 assay. The fluorescence of the SB216763 inhibitor was broad and overlapped with the green (540 nm) and red (595 nm) peaks of JC-1 emission (Figure 1A). This significantly affected the signals detected from the cells, as seen in Figure 1C. The microscopy images were corrupted by the fluorescence of the SB216763 inhibitor, and we were not able to conclude about the eventual changes of green/red intensities of JC-1. Fortunately, the JC-1 emission consisted of two well-separated peaks (Figure 1B). Each measured spectrum presented in Figure 1C is composed of these two JC-1 peaks and the SB216763 broad spectrum. The spectral deconvolution described in the Methods section enables reconstruction of the JC-1 spectrum without SB216763 interference, as presented in Figure 1D. With this heightened sensitive technique, the changes in the JC-1 spectra of the control, mock-treated cells indicated a gradual but measured depolarization over 75 min of analysis, in the absence of SB216763 (Figure 1E). In the presence of SB216763, depolarization was effectively inhibited (Figure 1E). These results suggest that GSK-3β activity is a prerequisite for the normal functioning of the mitochondrial permeability transition pore to open and close. Furthermore, we suggest inhibiting GSK-3β activity from influencing the dynamics of the mitochondrial transition pore can prevent the loss of ∆Ψ (Figure 1E).

Others have previously reported that SB216763 displays artifactual fluorescence [21]. The emission spectrum of SB216763-treated cells illustrates a broad spectrum over the range of 500–650 nm (Figure 1A). Furthermore, Figure 2 explicitly demonstrates the green fluorescence of undissolved SB216763 particulate matter with cells treated with SB216763 in the absence of annexin V-fluorescein isothicyanate/propidium iodide. The particulates stick to the cell surface of the cells and are not incorporated within cells. Moreover, the lack of red cells with a green halo in either the SB + annexin V-fluorescein isothicyanate/propidium iodide–treated cells (Figure 2, second row) or annexin V-fluorescein isothicyanate/propidium iodide–treated cells in the absence of SB216763 (Figure 2, third row) indicates that apoptosis/necrosis is not occurring under the conditions of the experiment with either the control or SB216763-treated cells. The latter point is not trivial as it confirms that the technical manipulation of switching the cells from hypoxia to oxygen exposure (refer to Methods) is not, of itself, sufficient oxidative stress to elicit entry into the cell death pathway.

The noted green fluorescent aggregates (Figure 2, fourth row) were likely due to the poor solubility of the inhibitor, and therefore, the inhibitor may not have been effective at inhibiting GSK-3β activity. To confirm that SB216763 did, indeed, inactivate GSK-3β activity, we treated the HLE-B3 cells with SB216763 and verified the enzymes’ inability to phosphorylate its downstream substrate, glycogen synthase (GS). The failure to adequately phosphorylate GS indicated the inactivation of the active site of GSK-3β (manuscript in preparation). The inhibition of GSK-3β activity (as monitored by the failure to phosphorylate GS) positively associated with preventing mitochondrial membrane permeability transition. We further examined the influence of the inactivation of GSK-3β activity on another substrate, β-catenin. β-catenin is a prominent nuclear transcription factor whose activity is regulated by GSK-3β. When GSK-3β is active, it inactivates β-catenin by phosphorylation. Treatment of HLE-B3 cells with SB216763 resulted in decreased phosphorylated β-catenin, the consequence of which was the increased translocation of β-catenin (active form) to the nucleus (data not shown).

In conclusion, the studies demonstrated that GSK-3β plays a pivotal role in regulating the mitochondrial membrane permeability transition pore under conditions of cell culture in atmospheric oxygen. Additionally, these studies have shown a novel technique that can be used to monitor changes in JC-1 fluorescence while in the presence of the drug SB216763.
